# Functions of the Nervous System—The Spinal Cord

**Published:** 1857-06

**Authors:** C. B. Chapman


### DENTAL REGISTER OF THE WEST.

VOL. X.—JUNE, 1857.—NO. 4.

### Original Essays and Communications.

### FUNCTIONS OF THE NERVOUS SYSTEM—THE SPINAL CORD.

BY PROF. C. B. CHAPMAN, M. D.

The nerve centres are as various in form as they are diverse in function. There is nothing analogous in form to the cerebrum elsewhere found, although there are other organs that as perfectly perform the function of a nerve centre, while that function is of a very different kind. The nerve centres, which belong to that group called the animal system, are the cerebrum—the cerebellum—the spinal cord and its appendage — the medulla oblongata, and the centres of the nerves of special sense. It was the aim of a former article to describe the centre, or organ of intelligence; it will be the object of this to present the main parts connected with the centre of sensation and motion, which has been variously called the excito-motor, and reflex centre.

The spinal cord occupies the spinal foramen, a canal which begins at the base of the cranium, by a large opening in the occipital bone called the foramen magnum, and extends through the whole group of bones that constitute the true vertebra, and a part of the false vertebra. It is inclosed in three layers of membrane, which correspond to those that envelop the cerebrum, and gives off a pair of nerves at the space between the several vertebral bones, and a pair above the first bone of the vertebra. The number of pairs of these nerves that pass out from the spinal cord are thirty-one, and their names correspond with that of the vertebral division from which they pass. The nerve fibres come out from two distinct portions of the spinal cord, or rather from four, when we remember that these nerves pass off laterally in pairs, each of which portion performs a distinct and independent function, (i. e.) the anterior and posterior division. The posterior half of the cord is separated from anterior by a deep fissure on each side, and the lateral portions are separated by a like fissure upon its posterior surface. The posterior columns constitute the centre of sensation, and so independent are they from each other, that one may be injured to an extent that will destroy its function without essentially impairing the use of the other.

The anterior half of the cord is as entirely appropriated to the function of motion, and the two sides are separated by a fissure on the anterior aspect, so as to render the function of the two halves, like those of the sensor columns, independent of each other, as well as of the posterior columns. Although these nerves are entirely different at their origin, as they come from three different portions of the spinal cord, their relations change as soon as they emerge from the intervertebral foramen, for there they mingle their fibres very freely, and then pass onward to be distributed to the most remote parts of the body. By thus commingling their fibres, the sources of which are so different, it is apparent that their two functions will be united in the same nerve cord or fibre, so that by exciting a nerve at its periphery, the impulse of sensation might be conveyed to the centre, (the spinal cord), and a part of the same cord imparts to the part or muscle, the power of voluntary motion. These thirty-one pairs of nerves, each divide and sub-divide into numerous branches, which are distributed to all parts of the body. So true is this, that there is no part that is not supplied with them; which fact can be demonstrated by an instrument of the most diminutive size, which cannot be made to puncture a soft part without injuring a nerve, and thus call the spinal cord into use.

The sensitive or posterior root may be distinguished from the anterior, or motor, by a small knot or ganglion which is situated upon it, just as it emerges from the intervertebral foramen, and just before the fibres from the two sources unite. There is another peculiarity in the co-mingling of nerve fibres, which renders their function more uniform, and secures a greater range of function from each; that is, by co-mingling of all the fibres of several nerves in a knot which is called a plexus. These are principally the cervical and axillary plexus, the first of which is formed by the union of six nerves into a broad knot. The fibres from each nerve which forms the plexus, contributes a small portion to make up each of the large number of nerves which pass around from the ganglion.

It will be perceived that great security against entire loss of sensation and power of motion is secured by this arrangement, for if the nerves which pass from one or more segments of the spinal cord, and contribute to form the plexus, should be injured or destroyed, while even one pair should remain uninjured, some degree of functional activity would be maintained.

In addition to the thirty one pairs of nerves that have the spinal cord for their centre, and which are so much alike, that a description of the function of one is an exact description of the function of the others; there are twelve, (some say nine) pairs of nerves that come off from the nerve centres within the cranium and pass out through the several foramen that are found in this group of bones.

Some of these nerves are nearly like those derived directly from the spinal cord in the possession of the excito-motor function, while some principally possess the functional qualities of the nerves derived from the posterior or sensor column, and others are like those of the anterior or motor column. Three of these cranial nerves differ from each other, and from all others. They are not endowed with sensation or the power of motion; but it is the office of each to preside over a special sense. They are the OLFACTORY, OPTIC, and AUDITORY. A fourth which is the nerve of taste, (the gustatory), originates like the others from the sensor column, being a branch of the minor division of the fifth pair. The whole group of these nerves is indicated by the following table, each of which will be separately referred to.

TABLE SHOWING CRANIAL NERVES.

1st. The Olfactory.
2d. The Optic.
3d. Motores Oculorum.
4th. Trochlearis Oculi, or Patheticus.
5th. Trifacial, or Trigeminus.
6th. Abducens Oculi.
7th. Facial, or Portio Dura.
8th. Auditory, or Portio Mollis.
9th. Glosso-Pharyngeal.
10th. Pneumo-gastric, or Par Vagum.
11th. Spinal Accessory.
12th. Hypo-Glossal, Myo-Glossal.

Spinal nerves, alike in function, are in number
8. Cervical.
12. Dorsal.
5. Lumbar.
6. Sacral.

I will briefly refer to each of these nerves in accordance with the arrangement of the table, although they will not thus be placed in groups according to their functions.

1st. *Olfactory.* Passes through the cribriform plate of the ethmoid bone by a large number of filaments, and is distributed upon the mucous membrane of the walls of the nasal cavity, as well as upon the spongy bones that line that cavity. It is the nerve which specially presides over the sense of smell, while the nerve that responds to the contact of irritants, is derived from an entirely different source.

2d. *Optic.* Passes out through the optic foramen, deep in the orbit. It is the largest of the cranial nerves, and is divided into numerous and attenuated fibres which are expanded upon the inner surface of the globe of the eye, contributing to form one of its coats, the retina.

3d. *Motores Oculorum*, is the motor nerve of four of the six muscles which move the eye-ball, these muscles are the superior, the inferior, and the internal rectus and the inferior oblique.

4th. *Trochlearis Oculi, or Patheticus*, is distributed to the superior oblique muscle.

5th. The *Trifacial*, unlike the two last, is the great sensitive nerve of the face. It is divided into three principal branches, viz: the *opthalmic* the *superior* and the *inferior maxillary* division. The two last are the source of the superior and the inferior dental nerves, while the opthalmic division contributes its quota, by supplying the incissor teeth of the superior maxillary. It is the minor division, the inferior maxillary that gives off a large branch to the tongue, to endow that organ with the special function of taste, the gustatory.

6th. The *Abducens Oculi*, endows the external rectus with the power of motion.

7th. *Facial, or Portio-dura*, is a motor nerve of the face, it passes through the parotid gland, and is distributed by numerous branches to the muscles of the face.

8th. *Auditory, or Portio Mollis*, is named on account of its extreme softness, and is distributed to the internal ear.

9th. *Glosso-Pharyngeal*, is principally distributed on the mucous surface of the fauces, and on the back part of the tongue, but it sends a branch forward on each side, a little beneath the most prominent lateral portion, which supplies the edges and inferior surface of the tip of the tongue, and inosculates with the gustatory, or lingual branch of the *fifth* pair.

It seems to be a pretty well established fact, that while the upper surface and tip of the tongue is supplied from the minor division of the *fifth*, that the base and sides and under surface of the tip is supplied by the *ninth.*

10th. The *Par Vagum, or Pneumo-gastric*, is mainly an afferent or sensitive nerve, giving this function to the stomach and lungs. There is no nerve of the whole animal system, that has attracted more attention from careful physiologists, and which extended investigations have resulted in such discordant conclusions. It seems, however, to be a well established fact, that although a sensitive nerve at its root, it receives fibres in its course which imparts to it a small degree of motor influence.

11th. The *Spinal Accessory*, unlike the par vagum, is mostly a motor nerve, while it inosculates in its course with the par vagum, from which it receives branches, and with the cervical plexus from which it receives fibres with both sensitive and motor functions.

12th. *Hypo-Glossal.* The last nerve of the cranial group which we have to consider, is the hypo-glossal. This nerve is distributed to the muscles of the tongue, to which it imparts the power of motion and articulation.

I do not claim that this article presents more than an imperfect view of this important physiological subject. I have only expected to present enough to lead the scientific practitioner of the dental art, to continue his investigations by the use of some of the more elaborate articles which are found in our best modern physiological authorities. It remains to refer to the peculiar and *partially* special function of some of the nerves already described; and then to review some facts and hypothesis, with regard to the combined function of the centres and the several nerves.

